# Increased radiosensitivity and radiation-induced apoptosis in SRC-3 knockout mice

**DOI:** 10.1093/jrr/rrt132

**Published:** 2013-12-05

**Authors:** Jie Jin, Yu Wang, Jin Wang, Yang Xu, Shilei Chen, Junping Wang, Xinze Ran, Yongping Su

**Affiliations:** 1 Institute of Combined Injury, State Key Laboratory of Trauma, Burns and Combined Injury, Chongqing Engineering Research Center for Nanomedicine, College of Preventive Medicine, Third Military Medical University, Chongqing 400038, China; 2 Department of Hematology, Daping Hospital, Third Military Medical University, Chongqing, China

**Keywords:** Radiosensitivity, SRC-3, knockout mice, hematopoiesis, apoptosis

## Abstract

Steroid receptor coactivator-3 (SRC-3), a multifunctional transcriptional coactivator, plays an important role in regulation of cell apoptosis in chemoresistant cancer cells. However, its role in radiation-induced apoptosis in hematopoietic cells is still unclear. In this study, we used SRC-3 knockout (SRC-3^-/-^) mice to assess the role of SRC-3 in radiation-induced hematopoietic injury *in vivo*. After a range of doses of irradiation, SRC-3^-/-^ mice exhibited lower counts of peripheral blood cells and bone marrow (BM) mononuclear cells and excessive BM depression, which resulted in a significantly higher mortality compared with wildtype mice. Moreover, BM mononuclear cells obtained from SRC-3^-/-^ mice showed a remarkable increase in radiation-induced apoptosis. Collectively, our data demonstrate that SRC-3 plays a role in radiation-induced apoptosis of BM hematopoietic cells. Regulation of SRC-3 might influence the radiosensitivity of hematopoietic cells, which highlights a potential therapeutic target for radiation-induced hematopoietic injury.

## INTRODUCTION

Steroid receptor coactivator-3 (SRC-3), also known as NCOA3, ACTR, p/CIP, RAC3 and TRAM-1, is a coactivator of nuclear receptors in the SRC family that also contains SRC-1 and SRC-2. SRC-3 can act as a coactivator and interact with nuclear receptors and other transcription factors to enhance their effects on target gene transcription [1]. Accumulating *ex vivo* studies indicate that SRC-3 plays an important role in the regulation of several physiological processes, including growth and development, sexual maturation, female reproductive function and energy metabolism. Additionally, overexpression of SRC-3 has been speculated to be associated with the initiation and/or progression of carcinoma [2]. SRC-3 was first reported to be amplified in breast cancer 1 (AIB1) in 1997 [3]. Subsequently, functional studies have revealed that SRC-3 is overexpressed and implicated in numerous aspects of cancer [4–5]. For instance, SRC-3 can promote cell proliferation for cancer initiation and growth, and it can also participate in anti-apoptosis enhancing chemotherapeutic resistance in many cancer cells [6]. An increasing number of research studies have highlighted the pivotal roles of SRC-3 in the chemoresistance of cancer cells via mediating anti-apoptosis signaling pathways. For example, an *in vitro* study revealed that inhibition of SRC-3 significantly reduced cell viability in prostate, lung and liver cancer cell lines by binding to special SRC-3 molecular inhibitor and depressing SRC-3 protein expression [7]. Additionally, downregulation of SRC-3 by siRNA led to increased sensitivity of cancer cells to cytotoxic agent-induced apoptosis through many signaling pathways, such as reduced nuclear factor kappa B (NF-κB)-mediated transcription, depressed expression of apoptosis inhibitor bcl-2, increased production of mitochondrial apoptotic factors (caspases-9 and caspases-7), enhanced AKT signaling and p38 kinase activities [8–10]. Moreover, further studies showed that SRC-3 possessed anti-apoptotic properties *in vivo* [11–12].

Recently, it has been reported that overexpression of SRC-3 in malignant blood cells could promote cell survival through inhibiting apoptosis [13–16]. For example, Colo *et al.* reported that SRC-3 was overexpressed in human chronic myeloid leukemia K562 cells, which were resistant to TRAIL-induced apoptosis [14]. Li *et al.* confirmed that SRC-3 played a role in anti-apoptosis in K562 cells by activating AKT signaling pathways [15]. Another study showed that downregulation of SRC-3 was involved in deguelin-induced apoptosis in Jurkat cells through inhibiting NF-κB target genes [16]. This suggests that SRC-3 could protect blood cells from cytotoxic agent-induced apoptosis. On the contrary, loss of SRC-3 increased the sensitivity of cell apoptosis after a chemical block. Although the mechanism of how SRC-3 is involved in hematopoietic regulation is still unclear, we propose that SRC-3 can affect cell survival and apoptosis in the hematopoietic system in the presence of an extrinsic stress.

It is well known that the hematopoietic system is a critical organ in radiation exposure. After a lethal dose of total body irradiation (TBI), hematopoietic cells in the bone marrow (BM) underwent apoptosis or radiation-induced mitotic death [17–18]. As a result, the number of hematopoietic cells decreased to an exhaustive level, after which leucopenia, erythropenia and thrombocytopenia were observed, which ultimately resulted in infection, hemorrhage and death. Since the existing clinical therapies have limited control over patient exposure to lethal doses of irradiation, reducing radiation-induced apoptosis of hematopoietic cells is thought to be a promising strategy with which to improve hematopoietic recovery and decrease mortality [19–20]. We hypothesize that SRC-3 might play a role in regulation of radiation-induced apoptosis in hematopoietic cells. In addition, we hypothesize that loss of SRC-3 will increase the sensitivity of hematopoietic cells to apoptosis and enhance BM damage induced by irradiation. In this study, using SRC-3^−/-^ mice as an animal model, we aim to investigate the role of SRC-3 in the survival, hematopoietic recovery, peripheral blood cell counts, and apoptotic rate of BM nucleated cells after irradiation.

## MATERIALS AND METHODS

### Animals

SRC-3^-/-^ mice were kindly provided by Prof. Jianming Xu (Molecular and Cellular Biology Laboratory, Baylor College of Medicine, Houston, USA). The SRC-3 mutant colony was maintained by interbreeding heterozygous pairs. The mice were on a mixed 129/SvEv × C57BL/6J background. Female SRC-3^-/-^ mice and wild-type (WT) counterparts (aged 8–10 weeks) were used in this experiment. Mice were provided with sterilized water and chow *ad libitum* in a pathogen-free animal facility. All experimental protocols were approved by the Animal Care Committees of the Third Military Medical University.

### Irradiation

Total body irradiation (TBI) was performed using ^60^Co γ-radiation (0.934 Gy/min) at room temperature. The mice were irradiated with lethal doses of 8 and 6 Gy, and a sublethal dose of 5 Gy.

### Mouse tail PCR

The genotype of mice was determined using PCR analysis according to the previous reference [21]. Briefly, sections (1–2 mm in length) of tail tip were lysed in 50 mM NaOH. Then 1 µl of the upper layer of the mouse tail lysate was added to 25 µl PCR reaction system. PCR amplification was performed in a 25 µl volume using a mouse tail direct PCR kit (KOD FX, Toyobo, Osaka, Japan), as per the manufacturer's instructions. The primers used during the proliferation are listed in Table 1. The amplification product was electrophoresed on a 1.5% agarose gel for 15 min with a voltage of 90 V. The images were visualized using a Gel Doc 2000 gel imaging system (Bio-Rad, USA).

**Table 1.** Primer sequences of PCR amplification

Genotype		Primer sequences
SRC-3^−/-^	primer 1	5′-GATGAGTGGACTAGGCGAAAGCTCT-3′
	primer 3	5′-GGCGATTAAGTTGGGTAACGCCAG -3′
SRC-3^+/+^	primer 1	5′-GATGAGTGGACTAGGCGAAAGCTCT-3′
	primer 2	5′-GCTGAGATTTGCAGAGATGAGCTC -3′
SRC-3^+/-^	primer 1	5′-GATGAGTGGACTAGGCGAAAGCTCT-3′
	primer 2	5′-GCTGAGATTTGCAGAGATGAGCTC -3′
	primer 3	5′-GGCGATTAAGTTGGGTAACGCCAG -3′

### Survival assay

Animal survival was monitored daily and reported as the percentage of animals surviving 30 d after radiation.

### Peripheral blood cell count

Peripheral blood was collected from the tail vein using a capillary tube, and then mixed with EDTA in a 1.5 ml tube. Subsequently, the complete blood cell count was analyzed using a Sysmex 800i (Sysmex Co. Ltd, Thailand) automated cell counter.

### Bone marrow nucleated cell count

BM nucleated cells were flushed from femurs with Iscove's modified Dulbecco's medium (IMDM, Hyclone, Logan, USA) supplemented with 2% fetal bovine serum (FBS, Gibco, Grand Island, USA). After erythrocytes were lysed and washed with PBS twice, the resuspended cells were mononuclear cells of mouse BM. The number of mononuclear cells was determined using a hemocytometer.

### Apoptosis assay for BM mononuclear cells

Three mice were sacrificed at 24 h after a dose of 6 Gy irradiation in each of the SRC-3^-/-^ and WT groups. Meanwhile, the same numbers of animals in the control groups were sacrificed. BM nucleated cells were harvested from both irradiated mice and normal mice as described above. The BM mononuclear cells were stained using an Annexin-V-FLUOS staining kit (Roche Applied Science, Penzberg, Germany). In brief, the cells were incubated with Annexin-V fluorescein (dilution 1:50) and propidium iodide (PI, dilution 1:50) in Hepes buffer for 15 min in the dark at room temperature. Subsequently, the double-stained mononuclear cells were analyzed immediately on a flow cytometer (Becton Dickinson, USA).

### Histology

For the histological analyses, three mice of each strain were sacrificed on Day 7 after irradiation. Then pathological tissues were obtained from the sternum of each mouse and stored in buffered formalin. Sections (5 µm in depth) were then embedded in paraffin and stained with hematoxylin-eosin (H&E). The stained slides were observed using a charge-coupled device (CCD) camera (Olympus, Tokyo, Japan) with a magnification of × 200.

### Statistical analysis

SPSS 11.0 software was used for the statistical analysis. Data were presented as mean ± standard deviation (SD). One-way analysis of variance (ANOVA) was used to compare the intergroup differences. *P* < 0.05 was considered statistically significant.

## RESULTS

### Genotyping results

**Fig. 1.**
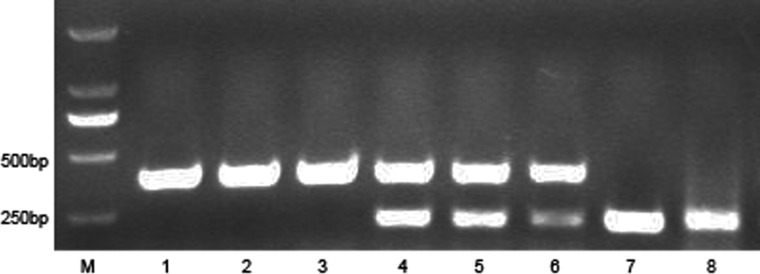
Genotyping results of SRC-3 mutant mice. Lane M: marker; Lanes 1–3: PCR results in SRC-3^+/+^ mice, a band of 450 bp was amplified using primer 1 and primer 2; Lanes 4–6: PCR results in SRC-3^+/-^ mice, two bands (450 bp and 230bp) were amplified using three primers; Lanes 7–8: PCR results in SRC-3^-/-^ mice, a band of 230 bp was amplified using primer 1 and primer 3.

The genotyping results are shown in **Fig. 1**. A 450-bp fragment was generated in SRC-3^+/+^ mice, while a 230-bp fragment (representing an allele of SRC-3 that was deleted) was amplified in SRC-3^-/-^ mice. To detect the heterozygote, both fragments of 450-bp and 230-bp were amplified with the three mixed primers from the mice tail DNA.

### Decreased survival rates in SRC-3^-/-^ mice after TBI

**Fig. 2.**
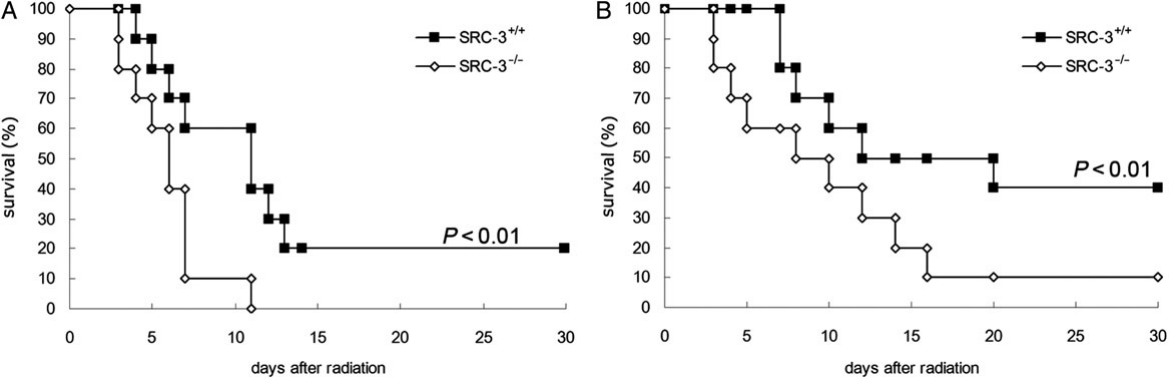
Survival rates of mice irradiated with 8 Gy **(A)** or 6 Gy **(B)** gamma-rays. SRC-3^-/-^ and WT mice were exposed to gamma rays of 8 Gy (A) or 6 Gy (B). Each treatment group included 10 mice. A significant difference was noticed in the survival rates between the SRC-3^-/-^ group and the WT group.

To evaluate the mortalities of SRC-3^-/-^ and WT mice caused by irradiation, we first observed the survival rates of mice exposed to lethal doses of 8 Gy or 6 Gy. No SRC-3^-/-^ mice survived for more than 11 d after a TBI of 8 Gy. On the contrary, 20% of WT mice survived 30 d after the same dose (*P* < 0.01, **Fig. 2A)**. After a 6 Gy dose of irradiation, a survival rate of 10% was observed on Day 16 in SRC-3^-/-^ mice, while for WT mice, the survival rate was 40% (*P* < 0.01, **Fig. 2B)**. In general, a significant decrease was noticed in the survival rates of SRC-3^-/-^ mice compared with the WT group after exposure to a TBI of 8 Gy or 6 Gy, respectively.

### Lower peripheral blood cells counts in SRC-3^-/-^ mice after TBI

**Fig. 3.**
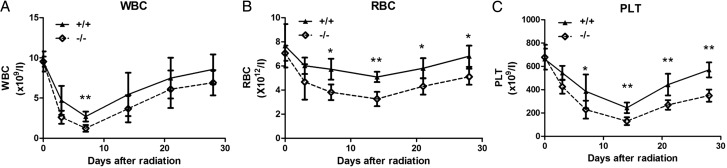
Peripheral blood cell counts in SRC-3^-/-^ and WT mice after a TBI of 5.0 Gy. The counts of WBCs **(A)**, RBCs **(B)** and PLTs **(C)** were significantly lower in SRC-3^-/-^ mice compared with those of WT mice. *P < 0.05; **P < 0.01.

It has been well known that the hematopoietic system is the major organ damaged by irradiation. To determine the hematopoietic injury caused by irradiation, the number of white blood cells (WBCs), red blood cells (RBCs) and platelets (PLTs) in peripheral blood was evaluated. After a sublethal dose of TBI (5 Gy), a marked decrease in WBCs, RBCs and PLTs was noted in WT and SRC-3^-/-^ mice. Compared with the WT mice, WBCs, RBCs and PLTs counts were lower in SRC-3^-/-^ mice from Day 1–30 after irradiation (**Fig. 3**). WBC counts decreased rapidly and reached a nadir on Day 7; the nadir of WBC in SRC-3^-/-^ mice was significantly lower than in WT mice (*P* < 0.01). The recovery rates of WBCs were nearly the same in both groups **(Fig. 3A)**. RBC counts decreased slowly to a nadir on Day 14 and recovered gradually, almost recovered in both groups by Day 28 post-irradiation; the RBC counts in SRC-3^-/-^ mice were observed to be significantly lower than in WT mice on Days 7, 14, 21 and 28 after TBI (*P* < 0.05) **(Fig. 3B)**. Similar to RBCs, there were significantly lower PLT counts in SRC-3^-/-^ mice compared with WT mice on Days 7, 14, 21 and 28 after TBI, and PLT counts in SRC-3^-/-^ mice reached pre-irradiation values of 56% (*P* < 0.01) **(Fig. 3C)**.

### Enhanced radiation effects on bone marrow in SRC-3^-/-^ mice after TBI

**Fig. 4.**
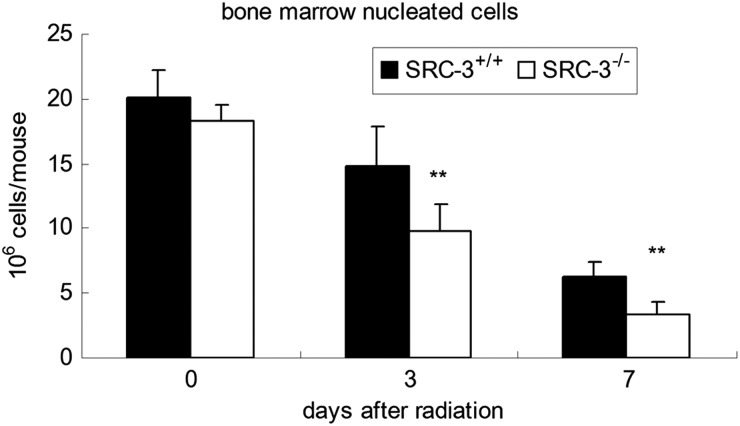
The cellularity of BM nucleated cells in SRC-3^−/-^ and WT mice on Day 3 and Day 7 after a TBI of 6.0 Gy. The number of nucleated cell numbers in SRC-3^−/-^ mice was significantly lower than that of WT mice on Day 3 and Day 7. *P < 0.05; **P < 0.01.

**Fig. 5.**
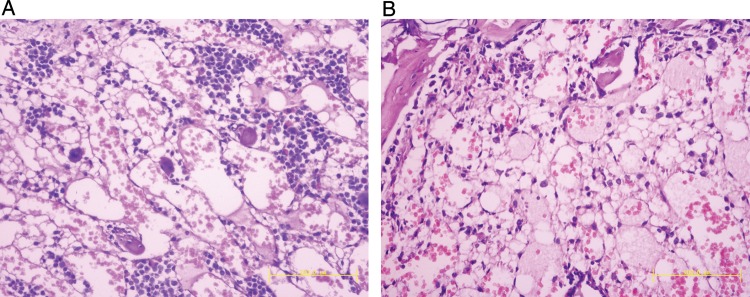
BM histological analyses of irradiated mice on Day 7 in WT mice **(A)** and SRC-3^−/-^ mice **(B)** exposed to a TBI of 6.0 Gy (*n* =3) (stained with hematoxylin and eosin, ×200).

To determine whether the increased mortality was caused by damage to mice BM induced by radiation, the number of BM nucleated cells was determined on Days 3 and 7 in WT and SRC-3^-/-^ mice after a TBI of 6 Gy **(Fig. 4)**. For the animals subjected to no irradiation, the BM cellularity in SRC-3^-/-^ mice was a little lower than in WT mice, but no statistical difference was noted between the two groups. After a 6-Gy irradiation, extreme reduction in the BM cellularity was noticed in both groups. Furthermore, the number of BM nucleated cells in SRC-3^-/-^ mice was significantly lower than that in WT mice on Days 3 and 7 after irradiation (*P* < 0.01), respectively. Consistent with the changes in BM nucleated cells, the number of hematopoietic cells showed a remarkable decrease in the SRC-3^-/-^ mice compared with the WT mice, as indicated by histological examination of the BM on Day 7 after irradiation **(Fig. 5)**.

### Increased apoptosis of BM mononuclear cells in SRC-3^-/-^ mice after irradiation

**Fig. 6.**
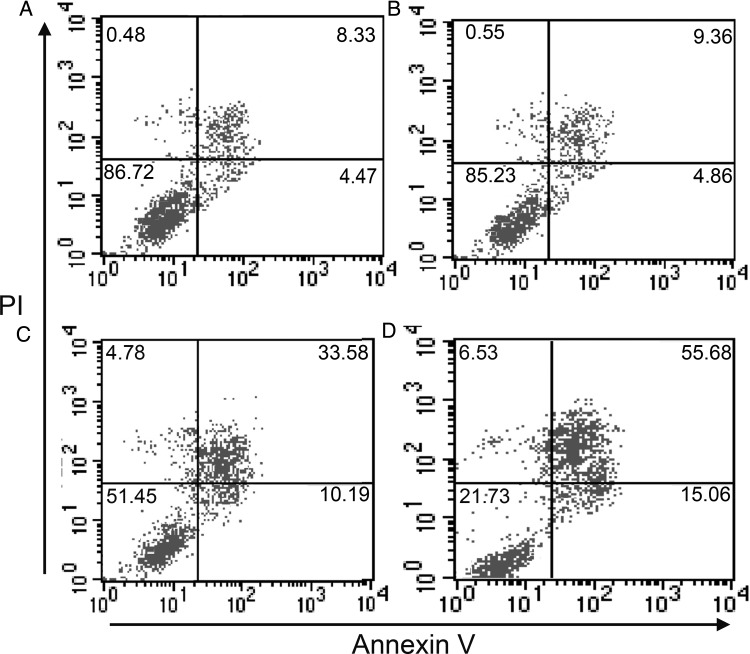
Apoptosis of BM nucleated cells in SRC-3^−/-^ and WT mice after 6 Gy irradiation and control (no irradiation). **(A)** Cells collected from WT mice exposed to no irradiation. **(B)** Cells collected from SRC-3^−/-^ mice exposed to no irradiation. **(C)** Cells collected from WT mice after irradiation. **(D)** Cells collected from SRC-3^−/-^ mice after irradiation. The percentages of early apoptotic cells (annexin-high/PI-low) and apoptotic/dead cells (annexin-high/PI-high) are shown for each cell population by flow cytometry.

It is clear that the hematopoietic injury caused by irradiation is thought to result from radiation effects on hematopoietic cells. Most hematopoietic cells presented apoptosis or mitotic death following irradiation. Figure 6 shows a comparison of apoptosis in BM mononuclear cells of SRC-3^-/-^ mice and WT mice. Before exposure to the irradiation, no statistical difference was noted in the apoptotic rates of mononuclear cells in SRC-3^-/-^ mice compared with the WT mice (9.36 ± 0.66% vs 8.33 ± 0.56%, *P* > 0.05). However, after a dose of 6 Gy irradiation, a statistical difference was observed in the apoptotic rate of mononuclear cells in SRC-3^−/-^ mice compared with that of the WT mice (33.58 ± 1.83% vs 55.68 ± 1.54, *P* < 0.01).

## DISCUSSION

In this study, we want to present the effects of radiation on radiosensitivity and apoptosis in SRC-3^-/-^ and WT mice. A series of indices were determined in SRC-3^-/-^ and WT mice after different doses of irradiation, including survival rates, peripheral blood cell counts, pathological changes in BM, and apoptotic rate of BM mononuclear cells. The results revealed that a remarkable increase in mortality and enhanced radiation-induced damages were observed in SRC-3^-/-^ mice as compared with the WT mice.

The first noteworthy finding of this study was that increased mortality was noticed in SRC-3^-/-^ mice compared with WT mice after exposure to a lethal dose of TBI of 8 Gy or 6 Gy, respectively. It has been generally accepted that hematopoietic system injury plays a critical role in radiation-induced death. The radiation-induced damage is considered to be associated with the radiation of BM hematopoietic cells. Consequently, we found that the BM cellularity in SRC-3^-/-^ mice was significantly lower on Days 3 and 7 compared with WT mice. Meanwhile, aplasia of SRC-3^-/-^ mice was observed on Day 7 after irradiation, as indicated by the histological results of BM. Additionally, the counts of peripheral blood cells were significantly lower in SRC-3^-/-^ mice than those obtained from the WT mice and, at the same time, the hematopoietic recovery was depressed to a greater extent in SRC-3^-/-^ mice compared with WT mice after a sublethal irradiation. These findings suggest greater damage to the hematopoietic system, contributing to a poorer survival rate, in the SRC-3^-/-^ mice. To further explain the mechanism of the decrease in hematopoietic cells in SRC-3^-/-^ mice, we tested the apoptotic rate of mononuclear cells in both SRC-3^-/-^ mice and WT mice *in vivo*. No statistical difference was noticed in the apoptotic rates of the hematopoietic cells between the SRC-3^-/-^ mice and the WT mice subjected to no irradiation. Nevertheless, after exposure to 6-Gy irradiation, the cell apoptotic rate in SRC-3^-/-^ mice was significantly higher than that in WT mice. These results suggest that SRC-3 plays a role in radiation-induced apoptosis of hematopoietic cells in mouse BM, as we hypothesized.

As is well known, ionizing radiation causes cell apoptosis. We are aware of many important functional factors involved in multiple signaling pathways during radiation-induced cell apoptosis. Among these, p53, a crucial factor involved in both hematopoiesis and radiation-induced apoptosis, has been validated as playing an important role in the proliferation and apoptosis of hematopoietic cells [22–23]. In addition, increased radioresistance has been noticed in both hematopoietic stem cells (HSCs) and hematopoietic progenitor cells (HPCs) in p53-deficient mice [24–25]. Interestingly, a recent study showed that SRC-3 was inversely correlated with p53-regulated cell resistance to cytotoxic stress in breast cancers [26]. Also, overexpression of SRC-3 induced a significant decrease in p53 protein levels in sodium nitroprusside (SNP)-treated MCF-7 cells. In contrast, a high level of apoptosis was observed in SRC-3-depleted cells, accompanied by a remarkable increase in p53 protein levels. Yi *et al.* concluded that SRC-3 could negatively regulate p53 function in cell apoptosis. Accordingly, we presume that SRC-3 may play a role in protecting hematopoietic cells from radiation-induced apoptosis through regulation of p53 function.

NF-κB signaling is another important signaling pathway involved in radiation and apoptosis [27–28]. The activation of the NF-κB signaling pathway is causally related to ionizing radiation, and implicated in cell survival and radioresistance through regulation of several cell signaling pathways simultaneously, including inducing the upregulation of anti-apoptotic proteins (e.g. c-FLIP, bcl-2, bcl-xl and bfl-1), inhibiting the expression of apoptotic proteins (e.g. bad and bax), modulating cell progression controllers (cyclin D1, cyclin B1), and activating the AKT signaling pathway [29–32]. Previous studies revealed that SRC-3 was able to activate the NF-κB signaling pathway in coordination with IκB kinase (IKK) and act as a transcriptional coactivator for NF-κB in cancer cells [33–34]. With regard to the role of SRC-3 in blood cancer cells, recent research has demonstrated that SRC-3 plays an important role in cytotoxic agent-induced apoptosis through regulation of NF-κB-mediated signaling pathways. Colo *et al.* found that inhibition of SRC-3 by siRNA increased TRAIL-mediated apoptosis in the human chronic myeloid leukemia cells K562 by depression of NF-κB basal activity [14]. Another study showed that downregulation of SRC-3 could improve the sensitivity of Jurkat cells to deguelin-induced apoptosis by inhibiting the target genes of NF-κB [15]. In our study, disruption of SRC-3 led to increased radiosensitivity in SRC-3^-/-^ mice. Additionally, the BM nucleated cells collected from SRC-3^-/-^ mice exhibited a higher apoptotic rate after exposure to irradiation compared with those collected from similarly treated WT mice. We propose that SRC-3 is involved in the coactivation of the NF-κB signaling pathway that contributes to regulation of the sensitivity of BM nucleated cells to radiation-induced apoptosis.

The multiple roles of SRC-3 as a transcriptional coactivator in various biological functions and in the development of carcinomas have been intensively studied, but no report has been carried out to investigate the roles of SRC-3 in cell viability and apoptosis under radiation stress. In this study, using SRC-3 knockout mice, we have for the first time demonstrated that SRC-3 caused multiple effects on the hematopoietic system after irradiation, both in whole body injury and cell apoptosis. Our results suggest that regulation of SRC-3 might influence the radiosensitivity of hematopoietic cells, highlighting a potential therapeutic target for radiation-induced hematopoietic injury.

## FUNDING

This work was supported by the National ‘863’ High-tech Development Plan (No. 2007AA02Z152), the National Natural Science Fund of China (No. 31071025) and the Special Fund from PLA (No. BSW11J009).
